# Long-term outcomes of endothelial keratoplasty in Chinese eyes at a University Hospital

**DOI:** 10.1186/s40662-014-0008-9

**Published:** 2014-12-01

**Authors:** Alvin L Young, Rachel PW Kwok, Vishal Jhanji, Lulu L Cheng, Srinivas K Rao

**Affiliations:** Department of Ophthalmology and Visual Sciences, The Chinese University of Hong Kong, Prince of Wales Hospital, Shatin, Hong Kong China; Darshan Eye Clinic, Chennai, India

**Keywords:** Endothelial keratoplasty, Keratoplasty, Long-term outcomes

## Abstract

**Background:**

Endothelial keratoplasty (EK) is used increasingly for the management of cases with endothelial dysfunction. Long-term outcomes of the surgery are not widely reported in the literature. We report our experience of EK in Chinese eyes at a University teaching hospital.

**Methods:**

Retrospective analysis was performed for all cases of EK performed between 2005 and 2009. Data analyzed included indication for surgery, associated surgical procedures, complications, best-corrected visual acuity (BCVA) and overall graft survival.

**Results:**

Overall, 22 eyes of 21 patients underwent EK (13 males, 8 females, mean age 71.8 ± 11.3 years). Pseudophakic bullous keratopathy was the leading indication for surgery (*n =* 12) followed by Fuchs’ endothelial dystrophy (*n =* 4), or both (*n =* 3). Other indications for surgery included failed penetrating keratoplasty (*n =* 2) and endothelial decompensation due to multiple surgeries (*n =* 1). Triple procedure was performed in 5 (22.7%) cases. Complications were noted in the form of postoperative interface hemorrhage (*n =* 2, 9%) and graft dislocation (*n =* 1, 4.5%). The mean postoperative endothelial cell density was 1069 ± 585.8 cells/mm^2^. The mean postoperative pachymetry was 675.8 ± 108.5 μm. The mean preoperative and postoperative intraocular pressure was 11.3 ± 3.2 and 13.9 ± 4.5 mmHg respectively. At the last follow-up (mean, 47.4 ± 13.7 months), BCVA was ≥20/70 in 9 (40.9%) cases. Causes of poor BCVA included primary graft failure (*n =* 4), graft decompensation (*n =* 4), advanced glaucoma (*n =* 2) and irreversible graft rejection (*n =* 2). Graft remained clear in 12 (54.5%) cases at the last follow-up. Average graft survival was 19.7 ± 17.7 months (median 17.5 months).

**Conclusions:**

Nearly half of the EK grafts in our study survived over a period of five years. Graft failure, glaucoma and graft rejection were the main causes of poor graft survival.

## Background

Endothelial keratoplasty (EK) has become the treatment of choice in cases with corneal endothelial dysfunction in the presence of clear corneal stroma. Over the past few years, the spectrum of indications of EK is expanding [1],[2]. It has been performed with successful outcomes in cases with bullous keratopathy, Fuchs’ endothelial dystrophy, iridocorneal endothelial syndrome, posterior polymorphous dystrophy and congenital hereditary endothelial dystrophy [3],[4]. Furthermore, as the refinements in the surgical techniques progress, new reports of intraoperative problems, postoperative issues and complications continue to surface [5]-[9]. EK has managed to come at par with penetrating keratoplasty, a surgery that was established more than a century ago. Clearly, of all the variants of EK, descemet stripping endothelial keratoplasty (DSEK) is the most popular, and widely practiced by corneal surgeons around the world. Small incision, few sutures, mild astigmatism and fast visual recovery have enabled DSEK to become the first-choice surgery in suitable cases. Although current literature is continually replenished with information on modifications of EK, long-term outcomes are still under-reported [10]-[15]. We adopted deep lamellar endothelial keratoplasty (DLEK) in the year 2005. However, it was quickly replaced by the more popular DSEK in 2006. In this study, we report the outcomes of EK in Chinese eyes from our centre over a period of five years. To our knowledge, there were no previous reports on long-term outcomes of EK in Chinese eyes.

## Methods

This was a retrospective case series of all cases of endothelial keratoplasties that were performed in the Corneal Unit of a university hospital between 2005 and 2009. Our tertiary care hospital caters to the ophthalmic needs of a population of about 1.3 million. We performed the first endothelial keratoplasty (DLEK) at our hospital in January 2005 according to a previously described technique [16]. DLEK was replaced by DSEK in the following year. Optical grade donor corneas with endothelial cell density >2000 cells/mm^2^ were used in all cases. All surgeries were performed using a standard surgical technique as described previously [17].

For DSEK, the donor was prepared manually on an artificial anterior chamber using lamellar dissectors. Briefly, a 5.5 mm scleral tunnel wound was created 2.0 mm behind the temporal limbus. The Descemet’s membrane was scored and stripped under viscoelastic cover. Viscoelastic was gently irrigated before the insertion of graft. A 7.5 mm donor endothelial lenticule was folded in a 60/40 taco and inserted in the anterior chamber with a pair of forceps. The anterior chamber was filled with air at the end of the surgery. Postoperatively, the patient was instructed to lie supine for two hours. Topical methylprednisolone 1% eye drops 6 times a day and levofloxacin 0.5% eye drops four times a day were prescribed for first six weeks postoperatively. Weekly follow-up was scheduled for the first four weeks. Subsequently, patients were followed every 4–6 weeks. Corticosteroid eye drops were tapered off to four times daily over the next three months. By the end of one year postoperatively, all cases were using twice daily corticosteroids. In cases with graft rejection, hourly prednisolone acetate 1% eye drops were started for the first 3 days. Treatment was adjusted according to the response.

Case records of patients who had undergone EK were retrieved from the medical records. Data analyzed included demographics, indication for surgery; type of EK performed, associated surgical procedures, complications, best-corrected visual acuity (BCVA) and overall graft survival. For the purpose of this study, graft survival was defined as the time period for which the graft remained clear after surgery. The study followed the tenets of the Declaration of Helsinki.

## Results

Over the study period, 22 eyes of 21 patients underwent EK (13 males, 8 females). Mean age of the patients was 71.8 ± 11.3 years. The indications for surgery are described in Table 1. Four cases underwent DLEK and 17 underwent DSEK. Cataract extraction with intraocular lens implantation was performed in 5 (22.7%) cases.

**Tabl`e 1:** **Indications for endothelial keratoplasty**

Indication for surgery	*n* = 22
Pseudophakic bullous keratopathy	12
Fuchs’ endothelial dystrophy	4
Pseudophakic bullous keratopathy and Fuchs’ endothelial dystrophy	3
Failed penetrating keratoplasty	2
Endothelial decompensation	1

Mean follow-up of all cases was 47.4 ± 13.7 months. At the last follow-up, 15 (68.2%) cases had improved BCVA compared to the preoperative level. BCVA was ≥20/70 in 9 (40.9%) and ≥20/50 in 6 (27.3%) patients. Causes of poor final BCVA included primary graft failure (*n =* 4), graft decompensation (*n =* 4), advanced glaucoma (*n =* 2) and irreversible graft rejection (*n =* 2).

Specular microscopy was not possible preoperatively because of media opacity due to bullous keratopathy. Preoperative pachymetry was not routinely performed in our cases. The mean postoperative endothelial cell density was 1069 ± 585.8 cells/mm^2^ (mean ± standard deviation). The average postoperative pachymetry was 675.8 ± 108.5 μm. The average preoperative and postoperative intraocular pressure was 11.3 ± 3.2 and 13.9 ± 4.5 mmHg respectively. Grafts remained clear in 12 (54.5%) cases at the last follow-up. Average graft survival was 19.7 ± 17.7 months (median 17.5 months).

Postoperatively, interface hemorrhage was observed in 2 (9%) cases. Although interface irrigation was performed, the hemorrhage reappeared shortly thereafter in both cases. Spontaneous resolution of hemorrhage was noted in one of these cases over a period of 4 months. Graft failure ensued in the other case. One case (4.5%) of 85-year-old female underwent DSEK triple procedure for Fuchs’ endothelial dystrophy. Although the surgery was uneventful, she suffered from graft dislocation in the immediate postoperative period. Surgical repositioning of the donor lenticule was undertaken in the operating room along with intracameral injection of iso-expansile mixture of SF6 (20%) resulting in successful re-attachment of the graft. Primary graft failure was seen in 4 (18.2%) cases. One of these cases had a retrocorneal membrane. Late graft failure was seen in 4 (18.2%) cases due to poor endothelial survival. Re-graft was performed in one case with failed penetrating keratoplasty. Two cases (9.1%) suffered from graft rejection within the first 6 months of surgery. Reversal of rejection episode could not be achieved despite intensive corticosteroid therapy and consequently, these cases suffered from graft failure.

## Discussion and conclusions

Endothelial keratoplasty involves the selective replacement of diseased corneal endothelium with a healthy partial-thickness posterior corneal lenticule. The outcomes of EK have improved over the past 5 years with the introduction of different techniques that focused on reducing the incision size, performing atraumatic graft insertion into the eye and preventing graft dislocation [1]. A few studies have analyzed the long-term outcomes of EK [10]-[15]. In a retrospective cohort study, Ang et al. [10] showed that percent endothelial cell loss was lower in EK compared with penetrating keratoplasty at up to 3 years after the surgery. In another retrospective, interventional case series, Li et al. [11] reported visual outcomes 3 years after EK. The authors reported a statistically significant trend toward improvement in the average best spectacle corrected visual acuity at postoperative month 6 and postoperative years 2 and 3 after EK. Price et al. [12] showed that the 3-year survival rate did not differ significantly between EK and penetrating keratoplasty procedures. In the present study, we describe the long-term outcomes of endothelial keratoplasty in Chinese eyes operated at a single centre over a period of 5 years. The most common indication for surgery was bullous keratopathy in our study. Although EK is being performed for a variety of conditions associated with diseased corneal endothelium, pseudophakic bullous keratopathy and Fuchs’ endothelial dystrophy remain the leading indications for EK [2]. This can be partly attributed to gradual increase in the number of cataract surgeries being performed all over the world.

Although there were no intraoperative problems encountered in any of the cases in our study, the postoperative course was complicated by graft dislocation in one case that underwent DSEK along with cataract extraction and intraocular lens insertion for Fuchs’ endothelial dystrophy. Reattachment of the donor lenticule was achieved by intracameral tamponade using isoexpansile gas mixture. Graft dislocation is one of the most common complications of EK seen in the immediate postoperative period. In a recent report by the American Academy of Ophthalmology, nearly 85% of the studies reported graft dislocation after EK and dislocation rates varied from 0% to 82% [5]. Main causes of graft dislocation include incomplete irrigation of viscoelastic, surgical trauma, and, rubbing or squeezing the eye in the early postoperative period [5]. EK graft dislocation is typically evident within the initial week after surgery. Successful reattachment can be achieved in most cases by injection of air or gas between the graft and the iris similar to that in our case.

Primary graft failure is the third most common complication of EK [5]. The incidence of primary graft failure in our study matches well with the reported rates of 0% to 29% in the published literature [5]. Primary graft failures represent a lack of endothelial function due to unhealthy tissue, unhealthy recipient circumstances or surgical technique. Primary graft failure in DSEK can also occur because of primary donor endothelial failure but is more often attributed to excessive endothelial cell trauma during the surgical procedure. A recent retrospective case–control study of Asian patients [18] who underwent EK analyzed the risk factors in cases of primary graft failure. The rate of primary graft failure was 4.8% in this study. Significant risk factors associated with primary graft failure included graft insertion using a folding technique and a small donor diameter [18]. In another retrospective analysis [12], the principal causes of graft failure or regraft within 3 years after EK were immunologic graft rejection (0.6%), endothelial decompensation (1.7%), and unsatisfactory visual or refractive outcome.

We also encountered graft rejection in 9.1% of cases in our study. A typical episode of graft rejection after EK resembles the endothelial graft rejection after a conventional penetrating keratoplasty. Rejection rates vary from 0% to 45.5% among reported studies with follow-ups ranging from 3 to 24 months [19]-[21]. Although most graft rejection episodes after EK can be controlled with increased corticosteroid use, some cases can progress to graft failure as seen in our study.

In our study, nearly half of the grafts were clear at the last follow-up. The main causes of poor graft survival were primary (*n =* 4) and late graft failure (*n =* 4), and advanced glaucoma (*n =* 2). The reported [5] extrapolated graft survival from 13 reviewed studies at 1 year or beyond after EK ranges from 55% to 100% with an average of 94% graft survival at 1 year. In a recent study, [11] a statistically significant trend toward improvement in visual acuity was reported up to 3 years after endothelial keratoplasty.

Limitations of the present study include a retrospective design and small sample size. Also, we do not have data on endothelial cell counts and corneal thickness after EK. Nevertheless, our study presents the long-term follow-up outcomes after EK in Chinese eyes. Similar studies from other centers would be useful in order to compare EK and penetrating keratoplasty over an extended follow-up period.

## References

[1] Young AL, Rao SK, Lam DS (2008). Endothelial keratoplasty: where are we?. Clin Experiment Ophthalmol.

[2] Price MO, Price FW (2010). Endothelial keratoplasty - a review. Clin Experiment Ophthalmol.

[3] Ghaznawi N, Chen ES (2010). Descemet’s stripping automated endothelial keratoplasty: innovations in surgical technique. Curr Opin Ophthalmol.

[4] Young AL, Rao SK, Cheng LL, Lam PTH (2008). Endothelial keratoplasty. Hong Kong J Ophthalmol.

[5] Lee WB, Jacobs DS, Musch DC, Kaufman SC, Reinhart WJ, Shtein RM (2009). Descemet’s stripping endothelial keratoplasty: safety and outcomes: a report by the American Academy of Ophthalmology. Ophthalmology.

[6] Young AL, Tam PM, Lau TT, Cheng LL, Rao SK, Lam PT (2009). Case of post Descemet stripping endothelial keratoplasty retrocorneal fibrous membrane. Clin Experiment Ophthalmol.

[7] Suh LH, Yoo SH, Deobhakta A, Donaldson KE, Alfonso EC, Culbertson WW, O’Brien TP (2008). Complications of Descemet’s stripping with automated endothelial keratoplasty: survey of 118 eyes at One Institute. Ophthalmology.

[8] Prasher P, Muftuoglu O, Hsiao ML, Bowman RW, Hogan RN, Mootha VV (2009). Epithelial downgrowth after descemet stripping automated endothelial keratoplasty. Cornea.

[9] Young AL, Jhanji V, Fan AH, Tam PM, Cheng LL, Rao SK (2012). Severe and recurrent interface hemorrhage after endothelial keratoplasty. Optom Vis Sci.

[10] Ang M, Mehta JS, Lim F, Bose S, Htoon HM, Tan D (2012). Endothelial Cell Loss and Graft Survival after Descemets Stripping Automated Endothelial Keratoplasty and Penetrating Keratoplasty. Ophthalmology.

[11] Li JY, Terry MA, Goshe J, Davis-Boozer D, Shamie N (2012). Three-year visual acuity outcomes after Descemet’s stripping automated endothelial keratoplasty. Ophthalmology.

[12] Price MO, Gorovoy M, Price FW, Benetz BA, Menegay HJ, Lass JH (2013). Descemet’s Stripping Automated Endothelial Keratoplasty: Three-Year Graft and Endothelial Cell Survival Compared with Penetrating Keratoplasty. Ophthalmology.

[13] Guerra FP, Anshu A, Price MO, Giebel AW, Price FW (2011). Descemet’s membrane endothelial keratoplasty: prospective study of 1-year visual outcomes, graft survival, and endothelial cell loss. Ophthalmology.

[14] Ratanasit A, Gorovoy MS (2011). Long-term results of Descemet stripping automated endothelial keratoplasty. Cornea.

[15] Ousley PJ, Terry MA (2005). Stability of vision, topography, and endothelial cell density from 1 year to 2 years after deep lamellar endothelial keratoplasty surgery. Ophthalmology.

[16] Terry MA (2007). Endothelial keratoplasty: clinical outcomes in the two years following deep lamellar endothelial keratoplasty (an American Ophthalmological Society thesis). Trans Am Ophthalmol Soc.

[17] Basak SK (2008). Descemet stripping and endothelial keratoplasty in endothelial dysfunctions: three-month results in 75 eyes. Indian J Ophthalmol.

[18] Ang M, Htoon HM, Cajucom-Uy HY, Tan D, Mehta JS (2011). Donor and surgical risk factors for primary graft failure following Descemet’s stripping automated endothelial keratoplasty in Asian eyes. Clin Ophthalmol.

[19] Jordan CS, Price MO, Trespalacios R, Price FW (2009). Graft rejection episodes after Descemet stripping with endothelial keratoplasty: part one: clinical signs and symptoms. Br J Ophthalmol.

[20] Wu EI, Ritterband DC, Yu G, Shields RA, Seedor JA (2012). Graft Rejection Following Descemet Stripping Automated Endothelial Keratoplasty: Features, Risk Factors, and Outcomes. Am J Ophthalmol.

[21] Li JY, Terry MA, Goshe J, Shamie N, Davis-Boozer D (2012). Graft rejection after Descemet’s stripping automated endothelial keratoplasty: graft survival and endothelial cell loss. Ophthalmology.

